# Health-related quality of life within agriculture in England and Wales: results from a EQ-5D-3L self-report questionnaire

**DOI:** 10.1186/s12889-022-13790-w

**Published:** 2022-07-20

**Authors:** Rebecca Wheeler, Matt Lobley

**Affiliations:** Centre for Rural Policy Research, University of Exeter, Lazenby House, Prince of Wales Road, Exeter, England EX4 4PJ

**Keywords:** Health-related quality of life, Mental health, Physical health, Agriculture, Farming, Wellbeing

## Abstract

**Background:**

Mental and physical health problems among the farming community are well documented but there is limited evidence regarding the overall health status of this population. This paper offers a unique insight into this issue through presenting the findings from a survey instrument, the EQ-5D-3L, which provides a standardised measure of health-related quality of life (HRQOL).

**Methods:**

We conducted the largest ever survey (*n* = 15,296) of people living and working in agriculture in England and Wales to gather baseline data on health and wellbeing within this community. The survey included an assessment of HRQOL through the use of the EQ-5D-3L self-report questionnaire. A variety of statistical approaches were used to test for significant associations between HRQOL and sub-group characteristics, including the Chi-square test for independence, the Kruskal-Wallis test, and Mann-Whitney U-test. Binary logistic regression models were also created to assess the influence of a set of respondent characteristics on the likelihood of respondents reporting health problems in the EQ-5D-3L.

**Results:**

24% of respondents reported problems with mobility; 4% reported problems with self-care; 21% reported problems with performing their usual activities; 52% reported problems with pain/discomfort; and 31% reported problems with anxiety/depression. The mean EQ-5D index value was 0.811 (median (Md) 0.796, interquartile range (IQR) 0.275). The mean self-rated health score (EQ-VAS) was 77.6 (SD 16.1) (Md 80.0, IQR 20). In general, holding other respondent characteristics equal, women reported fewer problems with mobility, self-care, performing usual activities and pain/discomfort than men, but more problems with anxiety/depression. HRQOL in the working-aged appears to be poorer among the survey population than the wider UK population.

**Conclusions:**

Results reveal concerning levels of physical and mental health problems, especially pain/discomfort and anxiety/depression, which appear to be more prevalent within our sample than within the wider UK population. There were important gender and age-related differences in both mental and physical health. Combatting these problems through targeted support is essential for the wellbeing of the farming community and the future sustainability of UK food production.

**Supplementary Information:**

The online version contains supplementary material available at 10.1186/s12889-022-13790-w.

## Background

There has long been concern over high rates of mental and physical health problems among agricultural populations across the world [1–3]. In the United Kingdom (UK), national data has consistently identified agriculture as one of the poorest performing sectors in terms of suicide and physical injury. Between 2016/17 and 2020/21 Agriculture, Forestry and Fishing had the highest rate of work-related musculoskeletal disorders, fatal injuries and non-fatal injuries of all sectors in Great Britain [4]. Latest estimates for England suggest that males in elementary agricultural occupations have a suicide risk 1.9 times higher, and those in skilled agricultural occupations 1.7 times higher, than the national average [5].

Research on these issues across different international contexts has identified a range of factors that contribute to poor health among farming populations. In the developed world, these include financial difficulties [6, 7]; pressures associated with paperwork, regulation and inspection [8, 9]; loneliness and isolation [10–12]; work demands/long working hours [13, 14]; impacts of climate change and extreme weather [15–17]; pesticide exposure [18, 19]; and animal disease [20, 21]; as well as social, cultural and practical barriers around seeking help for health issues [22–24]. Mental and physical health problems are clearly concerning for the wellbeing of the individual, but they can also have wider impacts on the farm business and family. They can, for instance, be difficult for close family members to deal with (10) and can lead to the neglect of business responsibilities such as animal welfare [25, 26].

The COVID-19 pandemic has helped highlight the role of farmers as essential workers. In addition, in the UK radical changes to the policy environment in which farmers operate are being implemented following withdrawal from the European Union (EU). High rates of health problems could inhibit the ability of individual farming businesses to adapt and plan for the future. A detailed analysis of the health and well-being status of the agricultural community is, therefore, more timely than ever.

In 2021 we conducted a survey of people living and working in agriculture (*n* = 15,296) to investigate the overall health status and quality of life of this population, identify common sources of stress and explore perceptions of current farm business performance, challenges and opportunities. This article presents results from one survey instrument, the EQ-5D-3L,^1^ which provides a standardised measure of health-related quality of life (HRQOL) [27]. The EQ-5D-3L comprises a descriptive system questionnaire and a visual analogue scale (EQ-VAS). The descriptive system asks the respondent to indicate whether they have no, some or extreme problems in relation to five health dimensions (mobility; self-care; performing usual activities; pain/discomfort; and anxiety/depression), thereby enabling focused analysis regarding these particular health issues. Responses to the descriptive system can also be converted into index values, which provide a single aggregated measure of health for each individual based on their reported level of problems with the five health dimensions. The index value is calculated “according to a set of [country or region-specific] weights that reflect, on average, people’s preferences about how good or bad the state is” [27] (p21) and represents the societal perspective on health. The EQ-VAS, on the other hand, requires the respondent to self-rate their health on a vertical visual scale (with 0 being the worst and 100 being the best health they can imagine) and, as such, represents the person’s own perspective on the overall state of their health. Analysis of the EQ-5D-3L responses in our survey provides a broad indication of HRQOL within the agricultural population of England and Wales, including sub-group variations according to age and gender.

## Methods

The primary methodology was a questionnaire survey (described below), which was distributed across the agricultural community in England and Wales for completion between January and April 2021. The research set out to remedy the paucity of data on the health and wellbeing of the agricultural community by establishing a baseline on this topic using standardised and replicable measures, including the EQ-5D-3L.^2^ A license to use the EQ-5D-3L questions was obtained from the EuroQol Research Foundation and all guidelines regarding the use and analysis of this instrument, as set out in the official user guide [27], were adhered to.

### Survey design

In order to explore the health and wellbeing status of the farming community, a self-completion questionnaire was designed to collect data on (in the order that follows): key characteristics of the respondent and their farm; mental wellbeing and physical health (including use of the EQ-5D-3L, Warwick-Edinburgh Mental Wellbeing Scale (WEMWBS)^3^ and General Anxiety Disorder-7 scale (GAD-7); relationships with others; and indicators of farm business performance.^4^

The questionnaire was piloted with 17 farmers and minor adjustments were made. The research was reviewed and approved by the College of Social Sciences and International Studies Research Ethics Committee at the University of Exeter.

### Survey sampling and distribution

The questionnaire was available in hardcopy form and online using the Qualtrics survey platform. As explained below, the paper version of the questionnaire was distributed using a variety of avenues, which means we are unable to calculate an overall response rate. Both versions of the questionnaire and all accompanying documents were available in Welsh as well as English.

A mailing list of 28,000 farms across England and Wales was purchased from a commercial company (Experian). The sample covered a range of farm types and provided a good geographical coverage (see the full survey report for further details [28] ). We drew on the Dillman Total Design Method [29] in an attempt to maximise the response rate, which entails sending out reminders and further copies of the questionnaire at planned intervals. The questionnaire was professionally printed in a user-friendly booklet format and posted out along with a covering letter, information sheet and a pre-paid return envelope. Non-responders were sent a reminder postcard 3 weeks after the initial distribution. Finally, a new questionnaire pack was sent out to remaining non-responders approximately 2 months after the initial distribution. Respondents who opted in were entered into a prize draw with the chance to receive one of three £50 vouchers. Recipients of the questionnaire were informed that additional copies could be requested for other members of the farm household and farm workers. The survey distribution was also supported by a number of agricultural stakeholders and the online version was promoted on social media (see Additional file 1 Appendix A for details).

The survey was distributed for completion between January and April 2021. As such, the research took place amid the COVID-19 pandemic, overlapping with national lockdowns in England and Wales,^5^ and the findings should be viewed carefully in light of this specific wider social context. We return to this point in the discussion.

### Data analysis

Data analysis was conducted using the statistical software programme SPSS. As suggested in the EQ-5D-3L user guide [27], responses to the EQ-5D-3L descriptive system were first simplified by dichotomising the EQ-5D levels into ‘no problems’ and ‘any problems’ so that frequencies of reported problems for each health dimension could be investigated. A variety of statistical approaches were then used to test for significant associations between responses to the EQ-5D-3L / EQ-VAS and sub-group characteristics, with a *p*-value of ≤0.05 considered statistically significant. The primary statistical test used in relation to the EQ-5D-3L descriptive data reported in this article was the Chi-square test for independence, which is a non-parametric technique appropriate for categorical and ordinal data. Non-parametric techniques - the Kruskal-Wallis test and Mann-Whitney U-test - were also used in relation to the continuous index values and EQ-VAS data, which had positively skewed distributions (mean index value = 0.81; mean EQ-VAS score = 77.6). Binary logistic regression models were created to assess the impact of a set of predictor variables (age, gender, respondent role, farm type, farm size and farm tenure) on the odds that respondents would report that they had problems with each of the five health dimensions.^6^

Results from the data analysis were then reviewed in relation to UK population norms [30] and considered in the context of previous research exploring mental and physical health issues in agriculture.

## Results

### Sample characteristics

A total of 15,296 survey responses were received. The key characteristics of the sample are shown in Table 1. Sample representativeness in comparison to the national farming population is difficult to ascertain, since national data relates to farm holdings rather than individuals (our survey data may include responses from more than one individual associated with a single farm). However, the relative distribution of different farm types, sizes, locations and types of farm tenure within our sample do appear to be broadly comparable to national data on the structure of agriculture in England and Wales (further details on this can be found in the full survey report [28]).

**Table 1:** Sample characteristics

	N	%
**Response format**		
Paper	13,575	89
Online	1721	11
Total	15,296	100
**Response language**		
English	15,217	99.5
Welsh	79	0.5
Total	15,296	100
**Respondent type **		
Sole/primary farmer	7500	49
Farming member of farm household	5084	34
Non-farming member of farm household	716	5.6
Farm employee (director/manager)	849	5.6
Farm employee (other worker)	224	1.5
Farm contractor	139	0.9
Retired or semi-retired	223	1.5
Other	436	2.9
Total	15,171	100
**Respondent gender**		
Male	11,513	76
Female	3487	23
Other	12	0.1
Prefer not to say	72	0.5
Total	15,084	100
**Respondent age**		
18-24	348	2.4
25-34	704	4.8
35-44	1044	7.1
45-54	2237	15
55-64	4405	30
65-74	3776	26
75+	2212	15
Total	14,726	100
**Farm type**		
Mixed	4569	30
Lowland Grazing Livestock	2640	17
LFA (e.g. upland) Grazing Livestock	2245	15
Cereals	1812	12
Dairy	1774	12
General Cropping	1001	6.6
Horticulture	400	2.6
Specialist Poultry	155	1.0
Specialist Pigs	87	0.6
Other	486	3.2
Total	15,169	100
**Farm size**		
Less than 20 ha (ha)	1191	8.0
20-49 ha	2194	15
50-99 ha	2992	20
100-199 ha	3797	26
200-499 ha	3254	22
500 ha+	1441	9.7
Total	14,869	100
**Farm tenure**		
Wholly/mostly owned	10,312	68
Wholly/mostly rented	2711	18
Mixed tenure	1949	13
N/A	133	0.9
Total	15,105	100
**Home region**		
East Midlands	1452	9.9
Eastern	1652	11
North East	540	3.7
North West & Merseyside	1437	9.8
South East (incl. London)	1486	10
South West	2872	20
Wales	1853	13
West Midlands	1507	10
Yorkshire & Humber	1604	11
Other	228	1.6
Total	14,631	100

NB: Totals for some characteristics differ from the full sample of 15,296 due to missing data, as not all respondents answered every question. All analysis has been conducted using valid percentages for the corresponding question(s).

Responses were received from across all adult age groups. The sample is skewed slightly towards the 55-64 and 65-74 age groups, which is to be expected given that the average age of farmers in the UK is 60 [31]. The mean age of survey respondents was also 60 years old (median 61, mode 60), although this varied by gender with female respondents being, on average, slightly younger than male respondents (with a mean age of 56 and 61 respectively). The larger proportion of males (76%) compared to females (23%) in the sample also broadly reflects the gender balance in the wider UK farming population, where 73% of those employed in agriculture, forestry and fishing are men and 27% are women [32].

High-level findings relating to i) each of the five health dimensions in the EQ-5D-3L descriptive system, ii) the EQ-5D index values and iii) the EQ-VAS are presented below. UK population norms [30], established pre-pandemic, are included alongside these results to provide a degree of contextualisation, although we must stress that caution must be taken in making direct comparisons due to the possible influence of the COVID-19 pandemic on the HRQOL reported by our respondents (this issue is examined more closely in the subsequent discussion).

It should also be noted that the composition of our sample differs from UK data with regard to a range of sociodemographic characteristics (i.e. age, gender, education level, employment status). For example, the median age of the UK population is 40 (compared to 61 in our sample) and the gender balance is 49% male and 51% female (compared to 76% male and 23% female in our sample), and these differences will influence relative levels or poor/good health within our overall sample. We have focused on providing results broken down by age to partially address this, but the sample remains structurally distinct from the national population. Comparisons are therefore intended to be only broadly illustrative of potential differences between the survey and wider population.

### EQ-5D-3L descriptive system

**Fig. 1:**
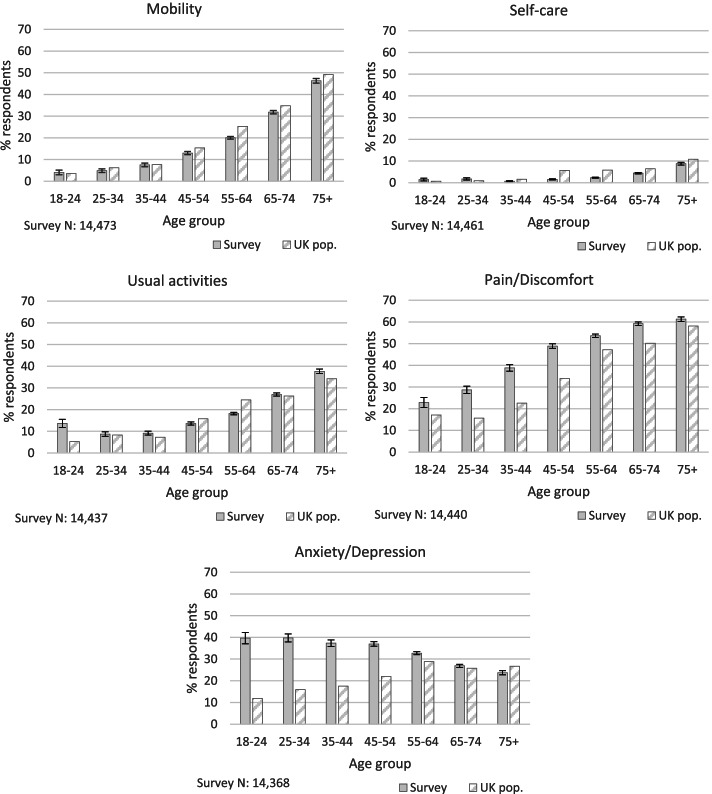
Reported problems (some or severe) with EQ-5D-3L health dimensions, agriculture survey and UK population (UK data source: [30]). Error bars show 95% confidence intervals

**Fig. 2:**
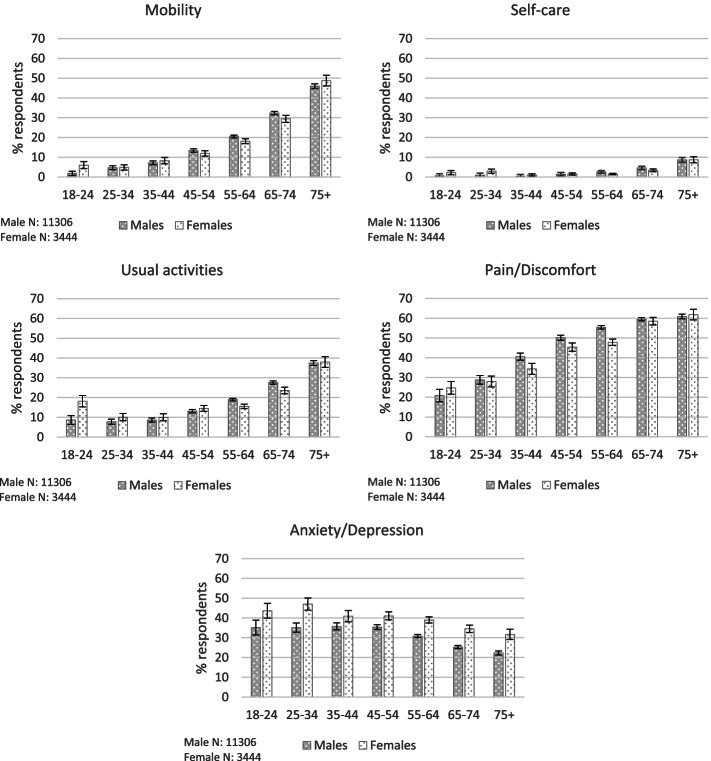
The association between reported health problems and gender. Error bars show 95% confidence intervals

Results for the EQ-5D-3L descriptive system are presented here in two levels (where ‘some’ problems are combined with ‘severe’ problems), since only small numbers of people reported severe problems (as is also usually the case in general populations [30]). Reported problems by all three levels can be found in Additional file 1 Appendix B. Results are presented by age (Fig. 1) and gender (Fig. 2) in order to elucidate key differences between sub-populations, whilst also taking into account differences in the age balance of the sample compared to UK data.

Binary logistic regression models assessing the influence of selected respondent characteristics (age, gender, respondent role, farm type, farm size and farm tenure) on the likelihood of reporting health problems confirmed that both age and gender had a unique and statistically significant effect for all the health dimensions. All models were found to be statistically significant (see Table 2). The key results from these models concerning gender are reported in the text below, as these highlight the relative influence of this factor whilst controlling for other respondent characteristics, including age (which is known to strongly influence health issues). Further results deriving from these models are not discussed in full here but can be viewed in Additional file 1 Appendix C (Tables C.1 – C.5).

**Table 2:** Significance figures for binary logistic regression models

	χ^2^	df	*N*	*p*	Percentage Correct
Mobility	1411.01	31	13,656	<.001	76.5
Self-care	268.30	31	13,625	<.001	96.6
Performing usual activities	786.23	31	13,645	<.001	78.9
Pain/discomfort	721.56	31	13,631	<.001	59.7
Anxiety/depression	309.92	31	13,565	<.001	68.7

### Mobility

24% of respondents reported having ‘some’ or ‘severe’ problems with mobility (defined as ‘walking about’) at the time of the survey. A Chi-Square Test for Independence indicated a significant association between mobility and age, with respondents aged under 65 less likely than expected (under the null hypothesis of no association), and those aged 65 or over more likely than expected, to report problems (χ^2^ (6, *n* = 14,473) = 1263.34, *p* < .001, Cramer’s V = .30).

Within each age group, a slightly lower proportion of respondents reported mobility problems compared to the UK population except in the 18-24 and 35-44 age groups where the difference in proportions was not statistically distinguishable from zero. The biggest difference was in the 55-64 age group, where 20% of respondents reported problems compared to 25% of the UK population.

Chi-Square Tests for Independence (with Yates’ Continuity Correction) indicated no statistically significant associations between gender and mobility problems. However, binary logistic regression revealed that, when all other respondent characteristics were held constant, males were around 1.12 (95% CI 1.01-1.26) times more likely than females to report problems with mobility (*p* = .036).

### Self-care

Reported problems with self-care were low, with just 4% of respondents reporting some or severe problems with this health dimension. A Chi-Square Test for Independence indicated a significant association between age and self-care problems, with respondents in age groups younger than 65 less likely than expected, and those in older age groups more likely than expected, to report problems (χ^2^ (6, *n* = 14,437) = 260.22, *p* < .001, Cramer’s V = .13).

Reported problems by age appear similar to those observed in the UK population. The biggest disparity was in the 45-54 age group, where 2% of respondents reported problems compared to 6% in the UK population.

Chi-Square Tests for Independence (with Yates’ Continuity Correction) indicated no statistically significant associations between gender and mobility problems. However, binary logistic regression revealed that, holding all other respondent characteristics constant, males were around 1.29 (95% CI 1.00-1.67) times more likely than females to report problems with self-care (*p* = .005).

### Usual activities

21% of respondents reported some or severe problems with performing their usual activities. A Chi-Square Test for Independence indicated a significant association between age and reported problems with performing usual activities (χ^2^ (6, *n* = 14,461) = 679.22, *p* < .001, Cramer’s V = .22). As with mobility and self-care problems, respondents younger than 65 were less likely than expected, and those in older age groups more likely than expected to report problems performing their usual activities.

Comparison with UK data indicates some differences between the agricultural and wider population in some age groups with regards to this health dimension. In particular, the proportion of respondents aged 18-24 reporting problems with usual activities was notably greater than in the UK population (14% compared to 5%). The proportion of respondents in the 75+ age group reporting problems also appears higher than in the wider population (38% compared to 34%). On the other hand, a smaller proportion of respondents aged 55-64 reported problems compared to the UK population (18% compared to 25%).

Chi-Square Tests for Independence indicated significant associations between gender and reported problems performing usual activities for some, but not all, age groups. Females aged 18-24 were significantly more likely than males of the same age to report problems with this health dimension (χ^2^ (1, *n* = 344) = 6.54, *p* = .011, Cramer’s V = .14), whereas males aged 55-64 (χ^2^ (1, *n* = 4283) = 6.003, *p* = .016, Cramer’s V = .04) and 65-74 (χ^2^ (1, *n* = 3642) = 4.33, *p* = .037, Cramer’s V = .04) were more likely than females of the same age to report problems. Overall, binary logistic regression revealed that, holding all other respondent characteristics constant, males were around 1.14 (95% CI 1.02-1.27) times more likely than females to report problems with performing usual activities (*p* = .022).

### Pain/discomfort

52% of respondents reported experiencing either ‘moderate’ or ‘extreme’ pain/discomfort at the time of completing the survey. A Chi-Square Test for Independence indicated a significant association between age and reported pain/discomfort (χ^2^ (6, *n* = 14,440) = 503.18, *p* < .001, Cramer’s V = .19). Respondents in age groups under 55 were less likely than expected, and those aged 65 and over more likely than expected, to report problems with this health dimension.

There is a notable disparity between our respondents and the wider UK population regarding levels of reported pain/discomfort across all age groups but particularly within the 25 to 54 year old age groups. For example, 39% of respondents aged 35-44 reported pain/discomfort compared to 23% of this age group within the general population.

Chi-Square Tests for Independence indicated a statistically significant association between gender and reported pain/discomfort for those aged 45-54 years old (χ^2^ (1, *n* = 2187) = 3.90, *p* = .48, Cramer’s V = .04) and 55-64 years old (χ^2^ (1, *n* = 4275) = 17.82, *p* < .001, Cramer’s V = .07), with males more likely than females to report problems, but there was no significant association with gender among the other age groups. Overall, binary logistic regression revealed that, holding all other respondent characteristics constant, males were around 1.22 (95% CI 1.12-1.34) times more likely than females to report problems with pain/discomfort (*p* < .001).

### Anxiety/depression

31% of all respondents reported some or severe problems with anxiety/depression at the time of completing the survey. A Chi-Square Test for Independence indicated a significant association between age and reported anxiety/depression (χ^2^ (6, *n* = 14,368) = 177.81, *p* < .001, Cramer’s V = .11). In contrast to the other health dimensions, it was the younger age groups (below 65) who were more likely than expected, and the older age groups (65 and above) less likely than expected, to report problems with this dimension. At least a third of respondents in all age groups between 18 and 64 reported being either ‘moderately’ or ‘extremely’ anxious or depressed.

There also appears to be considerable disparity in these age groups between our respondents and the wider UK population, particularly in those aged under 55, with higher proportions of those in agriculture reporting problems with anxiety/depression. For example, 40% of 18-24 year olds in our survey reported anxiety/depression compared with just 12% in the UK data.

Chi-Square Tests for Independence indicated a significant association between gender and anxiety/depression for all age groups except for 18-24 and 35-44 year olds (where there also appears to be associations but these were not statistically significant, *p* = .068 and .073 respectively), with females more likely than expected, and males less likely, to report problems with this health dimension (25-34 years old χ^2^ (1, *n* = 692) = 9.67, *p* = .002, Cramer’s V = .12; 45-54 years old χ^2^ (1, *n* = 2175) = 6.00, *p* = .014, Cramer’s V = .05; 55-64 years old χ^2^ (1, *n* = 4258) = 23.24, *p* < .001, Cramer’s V = .07; 65-74 years old χ^2^ (1, *n* = 3625) = 22.87, *p* < .001, Cramer’s V = .08; 75+ years old χ^2^ (1, *n* = 2046) = 13.26, *p* < .001, Cramer’s V = .08). Overall, binary logistic regression revealed that, holding all other respondent characteristics constant, males were around .70 (95% CI .64 to .76) less likely than females to report problems with anxiety/depression (*p* < .001).

### EQ-5D index values

Responses from the EQ-5D-3L descriptive system were converted into index values using a UK-specific TTO-based value set [33]. The mean index value for both the whole sample and for males only was 0.811 (Md 0.796, IQR 0.275). The mean index value for females was 0.814 (Md 0.814, IQR 0.275).

Kruskal-Wallis tests revealed that index values were significantly lower for respondents in age groups below 45 than for older age groups, for both males and females (male χ^2^ (6, *n* = 10,742) = *295*.84, *p* < .001; female χ^2^ (6, *n* = 3266) = 111.34, *p* < .001) (see Table C.6 in Additional file 1 Appendix C for sub-group n values).

**Fig. 3:**
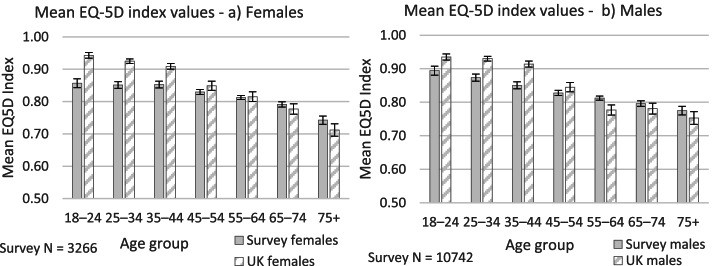
Mean EQ-5D index values (TTO value set), agriculture survey and UK population, **a**) Females and **b**) Males (UK data source: [30]). Error bars show 95% confidence intervals

For both males and females, mean index values appear to be lower than population norms for the UK for age groups below 55, but marginally higher for older age groups (see Fig. 3). For all *male* age groups, however, the differences between the mean index values for the survey and UK populations are less than 0.074 (the greatest difference being 0.064 for the 35-44 age group), which is the level identified by Walters and Brazier [34] as the minimal important difference (MID) for the EQ-5D-3L.^7^ For females, differences in mean index values between the survey and UK populations are similarly negligible for age groups over 35 years old, but equal the MID threshold for 25-34 year olds (0.074) and exceed it for 18-24 year olds (0.086).

### Self-rated heath scores (EQ-VAS)

**Fig. 4:**
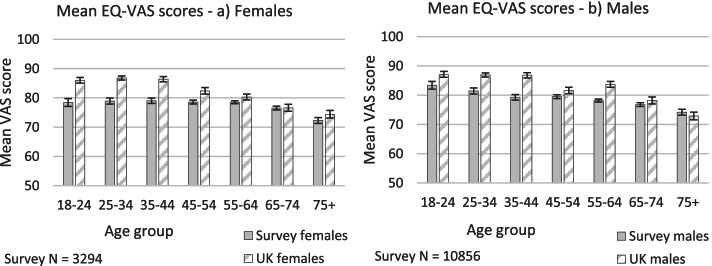
Mean self-rated health (EQ-VAS) scores, agriculture survey and UK population, **a**) Females and **b**) Males (UK data source: [30]). Error bars show 95% confidence intervals

The mean self-rated health score for all respondents was 77.6 (SD 16.1) (Md 80.0, IQR 20). The mean for the UK population is 82.8 [30]. These should not be directly compared due to the skewed age and gender distribution of our sample, however more detailed analysis does suggest that people living/working in agriculture were more likely to have a lower self-rated health score at the time of the survey compared to UK data (see Fig. 4). For both males and females, self-rated health within the 65-74 and 75+ years old age groups was similar to that within UK data. However, for those in age groups between 18 and 44 years old (and to a lesser extent those between 45 and 64), self-rated health was notably lower than in the UK data, particularly for females. The biggest disparities were in the 35-44 year old age groups, where the mean score for female respondents was 79.3 compared to 86.4 in the UK data, and the mean score for male respondents was 79.3 compared to 86.8 in the UK data.

Self-rated health was generally higher for younger respondents than older respondents. Kruskal-Wallis tests revealed a statistically significant association between self-rated health and age for both males and females (male χ^2^ (6, *n* = 10,856) = 182.60, *p* < .001; female χ^2^ (6, *n* = 3294) = 50.94, *p* < .0013) (see Table C.7 in Additional file 1 Appendix C for sub-group n values). Female respondents aged 75+ recorded significantly lower median scores (*Md* = 75) than those in younger age groups (age groups between 18 and 34 *Md* = 82; age groups between 35 and 74 *Md* = 80). Male respondents in age groups older than 35 years old recorded significantly lower median scores than those in the younger age groups (18-24 years old *Md* = 86, 25-34 years old *Md* = 85; 35-44 years old *Md* = 81; all age groups over 45 years old *Md* = 80).

## Discussion

As far as we are aware, this is the first time that the HRQOL of farmers, farm families and other agricultural workers in England and Wales has been assessed on a large scale. The results reveal concerning levels of reported health problems among people living and/or working in agriculture. The findings that over half (52%) of respondents reported having moderate or extreme pain/discomfort and almost a third (31%) reported some or severe problems with anxiety/depression at the time of completing the survey are striking. The timing of the survey (i.e. during the COVID-19 pandemic and partially in a period of national lockdown) to some extent restricts the conclusions that can be made about respondents’ health in relation to non-pandemic conditions or historical national population data, as it is possible that increased levels of social isolation and restricted access to routine healthcare at this particular point in time would have negatively impacted HRQOL. There are, however, a number of considerations that lead us to contend that such impacts are unlikely to account for all of the disparities observed between our sample and national data.

First, a growing body of literature has already highlighted significant mental and physical health problems among farmers both within the UK and internationally [3, 35–38] and has established links between these and a variety of personal, family and business-related challenges commonly faced by members of this community. The renowned high rates of both suicide and work-related injuries within agriculture compared to other occupations [1, 4, 39–41] are conspicuous indicators of such issues. Previous studies using the EQ-5D among farming populations have also found farmers to have lower EQ-5D index scores than non-farmers both at a local level within England [42] and elsewhere (e.g. Finland [43] and China [44].

Second (and in line with national health and safety statistics [4]), the findings from other questions included in our survey suggest that farm work has a negative impact on health. For example, 64% of respondents said that they had experienced farm-related ‘pain in muscles/joints etc.’, and 16% had sustained a non-fatal injury, in the past 5 years [28]. Respondents also cited a wide range of farming-related factors as significant sources of stress and there were significant associations between poor perceptions of economic performance/farm viability and low mental health [28]*.*

Finally, there are certain age-related patterns in our results that indicate unusually high levels of health problems among working-aged people in agriculture, which we examine further below.

The index values derived from the responses to the EQ-5D-3L descriptive system are lower than population norms within the age groups below 55 years old, indicating that these groups in our sample have a slightly lower health status than the general population from a societal perspective. The lower mean index values for females aged 18-24 and 25-34 compared to the wider population were particularly notable, exceeding the MID threshold established by Walters and Brazier [34] and suggesting that young female adults in agriculture deserve particular attention in efforts to improve HRQOL. Although the differences in index values observed for older females and all males in relation to the wider population were less significant (and even potentially positive in the case of the oldest age groups), the more notable variations in the 5 health dimensions indicate that the *types* of health issues experienced by farming are appreciably distinct and deserve further scrutiny. The results from the EQ-VAS indicate that self-perceptions of overall health are also poorer among our sample than among the wider population for both females and males, and this is the case across all except the oldest age groups (65 years old and over). It is notable that in both the index and VAS measures, disparities between the survey and UK populations were greatest among those of working age, which is when participation in farm work and business-related stress is likely to be at its most intense (both for primary farmers and other members of their families). Analysis of self-reported health problems from the EQ-5D-3L also revealed disparities between the survey sample and wider UK population among working-aged people in terms of i) pain/discomfort and ii) anxiety/depression, and it is plausible that the above health issues are at least partially influencing the lower levels of overall self-rated health. The gender analysis suggests that pain/discomfort is a particular issue for men, whereas anxiety/depression is a particular issue for women (although neither gender is immune from either health problem).

These findings are perhaps unsurprising when considered in the context of the nature of farming occupations. Farm work can involve considerable, at times intensive, manual labour and long-working hours with increased risks of physical injuries [41] and musculoskeletal disorders [14, 45, 46] that would be expected to lead to pain and discomfort. Notoriously long working hours and difficulties in taking time off from farm work [9] are only likely to exacerbate such health issues, as there is little opportunity for rest and recuperation. Although divisions of labour on farms are gradually changing [47], traditionally men have carried out more manual labour than women [48] and this might account for the higher levels of reported problems with pain/discomfort (and the other physical health dimensions) among men.

The high levels of self-reported anxiety/depression among working-aged people in our sample might similarly be explained by the likely occurrence of farm and business-related stress at this point in the life-course. Pressures and concerns associated with factors such as regulatory demands, paperwork, bad weather, disease, and maintaining economic viability may all be felt most acutely by those most involved in the everyday running of the farm. For working-aged women in farming in particular, these pressures are often accompanied by others relating to childcare responsibilities and/or involvement in diversified enterprises and off-farm work [10], with potential negative implications for mental health. Evidence suggests that the COVID-19 pandemic had a greater impact on mental health for younger compared to older people in the UK, particularly in terms of social isolation [49, 50], and this might have contributed to the heightened levels of anxiety/depression among this group. However, results from elsewhere in the survey suggest that it is unlikely to be the sole reason. Whilst COVID-19 was one of the top three sources of stress across the sample as a whole, it was more likely to be a source of significant stress for respondents over the age 65 (51% of 65-74 year olds and 53% of 75+ year olds 44% said it caused stress ‘quite a lot’ or ‘to a large extent’ compared to 44% of all respondents; (χ^2^ (6, *n* = 14,297) = 281.45, *p* < .001, Cramer’s V = .14)),. Furthermore, other stressors (including concerns about the future of the farm/farming, workload pressures/long working hours and financial pressures) were found to be better predictors of anxiety than the pandemic [28]*.*

More positively, respondents reported fewer problems with mobility and self-care than the wider population, particularly in the older age groups (45+). EQ-5D index values also appear higher than the wider population for those aged 75 and over. The reasons for this are, however, difficult to determine. It could suggest that farm work positively contributes to physical fitness later in life, keeping people active for longer, but it could also be a consequence of the requirement for a certain level of fitness in order to stay in agriculture (i.e. those with poorer mobility may already have left farming life). This demands further investigation. It is also interesting to note that, despite these better mobility levels, more respondents aged 75 or over (and to a lesser extent those aged 65-74) reported problems with performing their usual activities than the wider population. Given the tendency for farmers to retire (sometimes considerably) later than the state retirement age [51, 52], it is conceivable that this might be indicative of farmers trying to do more in their old age compared to their non-farming counterparts in spite of physical limitations. However, further research is required to affirm or refute such a supposition.

## Conclusions

The findings presented here serve as compelling evidence of the need to understand and address physical and mental health issues among people living and working in agriculture, as well as providing a baseline for future investigation into the health status of UK food producers. The results should be seen as an imperative for action as, ultimately, a sustainable and resilient food system requires healthy agricultural workers who are able to maintain and improve production without detriment to themselves and their families.

## Supplementary Information

**Additional file 1: Appendix A.** Additional information: methodology; **Appendix B.** Problems reported by dimensions (3 levels); **Appendix C.** Additional information: statistical tests.

## Data Availability

The datasets generated and/or analysed during the current study are not publicly available due to restrictions imposed by the funder but are available via the corresponding author on reasonable request.

## Abbreviations

Ha: Hectares
HRQOL: Health-related quality of life
IQR: Interquartile range
Md: Median
VAS: Visual analogue scale

## Footnotes

1 1990 EuroQol Research Foundation. EQ-5D™ is a trade mark of the EuroQol Research Foundation. UK(English) v2.1

2 The primary reason for selecting EQ-5D-3L rather than EQ-5D-5L was to manage the overall length of the questionnaire in order to minimise respondent fatigue and remain within our printing and postage budget. We also used shorter versions of other measures not reported on in this paper.

3 © NHS Health Scotland, the University of Warwick and University of Edinburgh, 2006, all rights reserved.

4 This paper does not report on the results of the WEMWBS or GAD-7 due to space restrictions but the results from these instruments reiterate the high levels of anxiety and depression that were identified through the EQ-5D-3 L and reported here. Further details relating to these instruments can be found in the survey report (28).

5 In England, the third national lockdown started on 6 January 2021 and restrictions began to be eased from 8 March 2021. In Wales, lockdown began on 26 December 2020 and restrictions began to be eased from 26 April 2021.

6 Note that, due to space limitations, only results relating to age and gender from the regression analysis are reported here.

7 The MID is the smallest difference in scores considered to be important enough to warrant a change in patient management from the clinical point of view (35).
