# Supplementary material for: Health-related quality of life within agriculture in England and Wales: results from a EQ-5D-3L self-report questionnaire
Source: BMC Public Health. 2022 Jul 20;22:1395. doi: 10.1186/s12889-022-13790-w (PMC9299404; doi:10.1186/s12889-022-13790-w)
Supplement: Supplementary file 1 — Additional file 1: Appendix A. Additional information: methodology; Appendix B. Problems reported by dimensions (3 levels); Appendix C. Additional information: statistical tests. [file 12889_2022_13790_MOESM1_ESM.pdf]

Appendix A. Additional information: survey distribution

The survey distribution was assisted by a number of agricultural stakeholders who, at their own cost or at a subsidised commercial rate, distributed paper copies of the questionnaire along with research ethics and consent information (return of a completed questionnaire was deemed to indicate consent). The following organisations/publications distributed the questionnaire and the Tenant Farmers Association sent the questionnaire to their members:

- National Farmers Union (NFU) – British Farmer & Grower magazine
- NFU – Student Farmer magazine
- NFU *Cymru* – Farming Wales magazine
- Farmers Weekly
- Farmers Guardian x 2
- Country Land and Business Association (CLA) – Land & Business Magazine

In addition, the online version of the survey was widely promoted on Twitter via the research team, RABI and a variety of farmer and stakeholder networks. This included a communication campaign by RABI (who have almost 10,000 Twitter followers), which included photos, quotes and videos by prominent people in the farming industry and was accompanied by the hashtag #BigFarmingSurvey. Nevertheless, the high proportion of paper responses we received was not unexpected, as we know (based on previous research experience and the advice of the RABI stakeholder group) that online questionnaires can be a challenge for farmers in rural areas with poor broadband and mobile network coverage.

Appendix B. Problems reported by dimensions (3 levels)

		Age														All	
		18-24		25-34		35-44		45-54		55-64		65-74		75+			
		n	%	n	%	n	%	n	%	n	%	n	%	n	%	n	%
Mobility	No problems	330	95.9	662	95.1	959	92.5	1927	87	3471	80	2531	68.2	1143	53.7	11023	76.2
	Some problems	14	4.1	34	4.9	77	7.4	288	13	864	19.9	1181	31.8	982	46.1	3440	23.8
	Confined to bed	0	0	0	0	1	0.1	0	0	5	0.1	1	0	3	0.1	10	0.1
Self-care	No problems	338	98.5	683	98.3	1030	99.3	2177	98.5	4233	97.7	3534	95.6	1934	91.2	13929	96.5
	Some problems	5	1.5	12	1.7	7	0.7	32	1.4	95	2.2	153	4.1	174	8.2	478	3.3
	Unable to	0	0	0	0	0	0	2	0.1	6	0.1	10	0.3	12	0.6	30	0.2
Usual activities	No problems	297	86.3	636	91.2	943	90.8	1912	86.4	3546	81.8	2704	73	1329	62.4	11367	78.6
	Some problems	46	13.4	59	8.5	89	8.6	291	13.1	762	17.6	951	25.7	762	35.8	2960	20.5
	Unable to	1	0.3	2	0.3	6	0.6	10	0.5	28	0.6	47	1.3	40	1.9	134	0.9
Pain/ discomfort	None	266	77.1	497	71.3	635	61.2	1131	51.1	2006	46.3	1509	40.8	822	38.7	6866	47.5
	Some	77	22.3	195	28	388	37.4	1049	47.4	2220	51.3	2091	56.5	1246	58.7	7266	50.3
	Extreme	2	0.6	5	0.7	15	1.4	32	1.4	102	2.4	98	2.7	54	2.5	308	2.1
Anxious/ depressed	No	207	60.3	420	60.3	648	62.7	1386	63	2900	67.3	2693	73.1	1603	76.3	9857	68.6
	Moderately	125	36.4	252	36.2	359	34.7	753	34.2	1323	30.7	956	26	471	22.4	4239	29.5
	Extremely	11	3.2	25	3.6	27	2.6	60	2.7	87	2	35	1	27	1.3	272	1.9

Appendix C. Additional information: statistical tests

Table C.1 Logistic regression analysis of reported problems with mobility

Predictor		B	S.E.	Wald	df	*p*	Odds Ratio	95% C.I. Lower	95% C.I. Upper
Age group	75+			947.364	6	0.000	1		
	18-24	-3.242	0.313	107.377	1	0.000	0.039	0.021	0.072
	25-34	-2.932	0.195	226.622	1	0.000	0.053	0.036	0.078
	35-44	-2.398	0.131	333.353	1	0.000	0.091	0.070	0.118
	45-54	-1.785	0.082	474.767	1	0.000	0.168	0.143	0.197
	55-64	-1.279	0.063	418.529	1	0.000	0.278	0.246	0.314
	65-74	-0.633	0.060	113.089	1	0.000	0.531	0.473	0.597
Gender	Female						1		
	Male	0.117	0.056	4.413	1	0.036	1.124	1.008	1.254
Farm type	Cereals			98.143	9	0.000	1		
	General cropping	0.023	0.109	0.046	1	0.830	1.024	0.827	1.268
	Horticulture	0.020	0.167	0.015	1	0.903	1.021	0.735	1.416
	Specialist Pigs	1.062	0.267	15.763	1	0.000	2.891	1.712	4.882
	Specialist Poultry	0.155	0.240	0.418	1	0.518	1.168	0.729	1.871
	Dairy	0.614	0.094	42.641	1	0.000	1.847	1.536	2.220
	LFA Grazing Livestock	0.645	0.087	55.076	1	0.000	1.906	1.608	2.260
	Lowland Grazing Livestock	0.418	0.088	22.836	1	0.000	1.519	1.280	1.804
	Mixed	0.405	0.078	27.243	1	0.000	1.499	1.288	1.746
	Other	0.533	0.136	15.431	1	0.000	1.703	1.306	2.222
Farm size	500+ ha			20.632	5	0.001	1		
	<20 ha	0.238	0.117	4.146	1	0.042	1.269	1.009	1.595
	20-49ha	0.292	0.099	8.638	1	0.003	1.339	1.102	1.627
	50-99ha	0.248	0.094	7.018	1	0.008	1.282	1.067	1.541
	100-199ha	0.105	0.090	1.360	1	0.244	1.111	0.931	1.326
	200-499ha	0.011	0.091	0.014	1	0.907	1.011	0.845	1.209
Farm tenure	Wholly/mostly owned			14.805	3	0.002	1		
	Wholly/mostly rented	0.189	0.058	10.831	1	0.001	1.208	1.080	1.353
	Mixed tenure	0.122	0.068	3.200	1	0.074	1.129	0.988	1.291
	N/A	0.624	0.356	3.072	1	0.080	1.866	0.929	3.747
Respondent role	Retired/semi-retired			2.075	7	0.956			
	Sole/primary farmer	-0.059	0.163	0.130	1	0.719	0.943	0.684	1.299
	Farming member of farm household	-0.062	0.165	0.142	1	0.706	0.940	0.680	1.299
	Non-farming member of farm household	-0.096	0.188	0.264	1	0.607	0.908	0.629	1.311
	Farm employee (director/manager)	-0.125	0.191	0.428	1	0.513	0.883	0.608	1.282
	Farm employee (any other worker)	-0.184	0.286	0.413	1	0.520	0.832	0.476	1.457
	Farm contractor	-0.103	0.305	0.113	1	0.736	0.903	0.497	1.640
	Other	0.077	0.206	0.139	1	0.709	1.080	0.721	1.617
				χ^2	df	*p*			
Overall model evaluation				1411.008	31	0.000			

Table C.2 Logistic regression analysis of reported problems with self-care

Predictor		B	S.E.	Wald	df	*p*	Odds Ratio	95% C.I. Lower	95% C.I. Upper
Age group	75+			173.714	6	0.000	1		
	18-24	-1.892	0.470	16.175	1	0.000	0.151	0.060	0.379
	25-34	-1.908	0.352	29.366	1	0.000	0.148	0.074	0.296
	35-44	-2.694	0.420	41.068	1	0.000	0.068	0.030	0.154
	45-54	-1.775	0.201	78.186	1	0.000	0.169	0.114	0.251
	55-64	-1.367	0.137	100.125	1	0.000	0.255	0.195	0.333
	65-74	-0.722	0.118	37.375	1	0.000	0.486	0.385	0.612
Gender	Female						1		
	Male	0.255	0.131	3.780	1	0.052	1.290	0.998	1.668
Farm type	Cereals			14.443	9	0.107			
	General cropping	0.178	0.248	0.514	1	0.473	1.194	0.735	1.941
	Horticulture	-0.032	0.377	0.007	1	0.933	0.969	0.463	2.028
	Specialist Pigs	0.636	0.554	1.317	1	0.251	1.890	0.637	5.602
	Specialist Poultry	-1.458	1.025	2.025	1	0.155	0.233	0.031	1.734
	Dairy	0.542	0.216	6.288	1	0.012	1.719	1.126	2.625
	LFA Grazing Livestock	0.502	0.200	6.285	1	0.012	1.652	1.116	2.446
	Lowland Grazing Livestock	0.362	0.199	3.323	1	0.068	1.437	0.973	2.122
	Mixed	0.271	0.184	2.179	1	0.140	1.311	0.915	1.879
	Other	0.153	0.309	0.247	1	0.619	1.166	0.637	2.135
Farm size	500+ ha			12.994	5	0.023	1		
	<20 ha	0.754	0.265	8.082	1	0.004	2.127	1.264	3.577
	20-49ha	0.473	0.241	3.858	1	0.050	1.604	1.001	2.571
	50-99ha	0.403	0.232	3.019	1	0.082	1.496	0.950	2.356
	100-199ha	0.388	0.224	2.995	1	0.084	1.474	0.950	2.288
	200-499ha	0.086	0.233	0.137	1	0.711	1.090	0.691	1.720
Farm tenure	Wholly/mostly owned			9.745	3	0.021	1		
	Wholly/mostly rented	0.222	0.130	2.911	1	0.088	1.249	0.967	1.612
	Mixed tenure	0.088	0.159	0.304	1	0.581	1.092	0.800	1.490
	N/A	1.369	0.510	7.206	1	0.007	3.931	1.447	10.680
Respondent role	Retired/semi-retired			7.181	7	0.410	1		
	Sole/primary farmer	-0.096	0.304	0.100	1	0.751	0.908	0.500	1.648
	Farming member of farm household	-0.080	0.310	0.066	1	0.797	0.923	0.503	1.694
	Non-farming member of farm household	0.120	0.353	0.114	1	0.735	1.127	0.564	2.253
	Farm employee (director/manager)	-0.175	0.395	0.196	1	0.658	0.840	0.387	1.820
	Farm employee (any other worker)	0.607	0.499	1.476	1	0.224	1.834	0.689	4.881
	Farm contractor	-0.665	0.785	0.717	1	0.397	0.514	0.110	2.396
	Other	0.322	0.385	0.699	1	0.403	1.380	0.649	2.936
				χ^2	df	*p*			
Overall model evaluation				268.298	31	<.001			

Table C.3 Logistic regression analysis of reported problems with performing usual activities

Predictor		B	S.E.	Wald	df	*p*	Odds Ratio	95% C.I. Lower	Upper
Age group	75+			549.635	6	0.000	1		
	18-24	-1.455	0.179	66.298	1	0.000	0.233	0.164	0.331
	25-34	-1.921	0.153	158.572	1	0.000	0.146	0.109	0.197
	35-44	-1.809	0.122	218.413	1	0.000	0.164	0.129	0.208
	45-54	-1.378	0.082	282.993	1	0.000	0.252	0.215	0.296
	55-64	-1.036	0.064	258.738	1	0.000	0.355	0.313	0.403
	65-74	-0.515	0.062	69.972	1	0.000	0.597	0.529	0.674
Gender	Female						1		
	Male	0.129	0.056	5.218	1	0.022	1.137	1.018	1.270
Farm type	Cereals			64.801	9	0.000	1		
	General cropping	0.084	0.110	0.576	1	0.448	1.087	0.876	1.350
	Horticulture	0.298	0.162	3.383	1	0.066	1.347	0.981	1.851
	Specialist Pigs	0.911	0.270	11.391	1	0.001	2.488	1.465	4.223
	Specialist Poultry	0.181	0.246	0.540	1	0.462	1.198	0.740	1.940
	Dairy	0.383	0.096	15.787	1	0.000	1.466	1.214	1.770
	LFA Grazing Livestock	0.615	0.088	49.060	1	0.000	1.851	1.558	2.198
	Lowland Grazing Livestock	0.361	0.089	16.448	1	0.000	1.435	1.205	1.708
	Mixed	0.297	0.079	14.059	1	0.000	1.346	1.152	1.572
	Other	0.480	0.139	11.884	1	0.001	1.616	1.230	2.123
Farm size	500+ ha			23.767	5	0.000	1		
	<20 ha	0.243	0.121	3.993	1	0.046	1.275	1.005	1.617
	20-49ha	0.394	0.103	14.622	1	0.000	1.484	1.212	1.816
	50-99ha	0.409	0.097	17.635	1	0.000	1.505	1.244	1.822
	100-199ha	0.302	0.094	10.340	1	0.001	1.352	1.125	1.625
	200-499ha	0.196	0.095	4.289	1	0.038	1.217	1.011	1.465
Farm tenure	Wholly/mostly owned			17.337	3	0.001	1		
	Wholly/mostly rented	0.181	0.058	9.735	1	0.002	1.199	1.070	1.344
	Mixed tenure	0.184	0.068	7.214	1	0.007	1.202	1.051	1.374
	N/A	0.679	0.348	3.805	1	0.051	1.972	0.997	3.900
Respondent role	Retired/semi-retired			5.742	7	0.570	1		
	Sole/primary farmer	-0.088	0.167	0.276	1	0.600	0.916	0.661	1.271
	Farming member of farm household	-0.109	0.169	0.419	1	0.518	0.897	0.644	1.248
	Non-farming member of farm household	-0.220	0.193	1.300	1	0.254	0.803	0.550	1.171
	Farm employee (director/manager)	-0.239	0.195	1.501	1	0.220	0.787	0.537	1.154
	Farm employee (any other worker)	0.158	0.257	0.378	1	0.539	1.171	0.708	1.939
	Farm contractor	-0.250	0.314	0.633	1	0.426	0.779	0.421	1.440
	Other	-0.112	0.212	0.279	1	0.597	0.894	0.590	1.355
				χ^2	df	*p*			
Overall model evaluation				786.234	31	<.001			

Table C.4 Logistic regression analysis of reported problems with pain or discomfort

Predictor		B	S.E.	Wald	df	*p*	Odds Ratio	95% C.I. Lower	Upper
Age group	75+			404.362	6	0.000	1		
	18-24	-1.687	0.145	135.764	1	0.000	0.185	0.139	0.246
	25-34	-1.393	0.102	188.005	1	0.000	0.248	0.204	0.303
	35-44	-0.927	0.083	125.476	1	0.000	0.396	0.337	0.466
	45-54	-0.528	0.066	63.292	1	0.000	0.590	0.518	0.672
	55-64	-0.329	0.058	31.899	1	0.000	0.720	0.642	0.807
	65-74	-0.099	0.059	2.792	1	0.095	0.906	0.807	1.017
Gender	Female						1		
	Male	0.202	0.044	20.549	1	0.000	1.223	1.121	1.335
Farm type	Cereals			104.309	9	0.000			
	General cropping	0.021	0.084	0.061	1	0.804	1.021	0.865	1.205
	Horticulture	-0.025	0.127	0.039	1	0.843	0.975	0.760	1.251
	Specialist Pigs	0.630	0.244	6.666	1	0.010	1.878	1.164	3.030
	Specialist Poultry	0.079	0.187	0.179	1	0.672	1.083	0.750	1.563
	Dairy	0.529	0.075	50.179	1	0.000	1.697	1.466	1.964
	LFA Grazing Livestock	0.511	0.070	53.105	1	0.000	1.667	1.453	1.912
	Lowland Grazing Livestock	0.379	0.070	28.966	1	0.000	1.460	1.272	1.677
	Mixed	0.367	0.060	37.205	1	0.000	1.444	1.283	1.625
	Other	0.469	0.118	15.930	1	0.000	1.599	1.270	2.013
Farm size	500+ ha			27.150	5	0.000	1		
	<20 ha	0.375	0.095	15.391	1	0.000	1.455	1.206	1.754
	20-49ha	0.398	0.080	24.759	1	0.000	1.488	1.273	1.741
	50-99ha	0.260	0.074	12.368	1	0.000	1.297	1.122	1.500
	100-199ha	0.283	0.070	16.440	1	0.000	1.327	1.157	1.521
	200-499ha	0.232	0.070	11.182	1	0.001	1.262	1.101	1.446
Farm tenure	Wholly/mostly owned			21.053	3	0.000	1		
	Wholly/mostly rented	0.157	0.047	10.957	1	0.001	1.170	1.066	1.284
	Mixed tenure	0.175	0.055	10.083	1	0.001	1.191	1.069	1.327
	N/A	0.652	0.319	4.175	1	0.041	1.920	1.027	3.588
Respondent role	Retired/semi-retired			18.272	7	0.011	1		
	Sole/primary farmer	0.101	0.161	0.398	1	0.528	1.107	0.808	1.516
	Farming member of farm household	0.019	0.162	0.013	1	0.908	1.019	0.742	1.399
	Non-farming member of farm household	-0.230	0.178	1.667	1	0.197	0.794	0.560	1.127
	Farm employee (director/manager)	-0.072	0.176	0.169	1	0.681	0.930	0.658	1.314
	Farm employee (any other worker)	0.047	0.222	0.045	1	0.832	1.048	0.678	1.620
	Farm contractor	0.028	0.258	0.012	1	0.914	1.028	0.620	1.704
	Other	0.019	0.192	0.010	1	0.922	1.019	0.699	1.486
				χ^2	df	*p*			
Overall model evaluation				721.563	31	<.001			

Table C.5 Logistic regression analysis of reported problems with anxiety or depression

Predictor		B	S.E.	Wald	df	*p*	Odds Ratio	95% C.I. Lower	Upper
Age group	75+			118.987	6	0.000	1		
	18-24	0.646	0.131	24.466	1	0.000	1.907	1.477	2.463
	25-34	0.677	0.100	45.957	1	0.000	1.969	1.619	2.395
	35-44	0.632	0.088	51.920	1	0.000	1.882	1.584	2.235
	45-54	0.588	0.073	64.823	1	0.000	1.800	1.560	2.076
	55-64	0.424	0.065	42.040	1	0.000	1.528	1.344	1.737
	65-74	0.180	0.067	7.172	1	0.007	1.197	1.049	1.365
Gender	Female						1		
	Male	-0.359	0.046	61.481	1	0.000	0.698	0.638	0.764
Farm type	Cereals			18.563	9	0.029	1		
	General cropping	0.127	0.094	1.848	1	0.174	1.136	0.945	1.364
	Horticulture	0.027	0.138	0.038	1	0.845	1.027	0.784	1.347
	Specialist Pigs	0.779	0.242	10.389	1	0.001	2.179	1.357	3.498
	Specialist Poultry	0.139	0.198	0.498	1	0.480	1.150	0.780	1.693
	Dairy	0.163	0.080	4.133	1	0.042	1.177	1.006	1.378
	LFA Grazing Livestock	0.133	0.076	3.043	1	0.081	1.142	0.984	1.326
	Lowland Grazing Livestock	0.133	0.077	3.021	1	0.082	1.142	0.983	1.328
	Mixed	0.203	0.066	9.335	1	0.002	1.225	1.075	1.395
	Other	0.069	0.127	0.297	1	0.586	1.072	0.836	1.374
Farm size	500+ ha			20.320	5	0.001	1		
	<20 ha	0.301	0.102	8.678	1	0.003	1.352	1.106	1.652
	20-49ha	0.345	0.086	15.922	1	0.000	1.412	1.192	1.672
	50-99ha	0.298	0.081	13.659	1	0.000	1.347	1.150	1.577
	100-199ha	0.273	0.076	12.913	1	0.000	1.314	1.132	1.526
	200-499ha	0.171	0.076	5.029	1	0.025	1.186	1.022	1.377
Farm tenure	Wholly/mostly owned			17.930	3	0.000	1		
	Wholly/mostly rented	0.191	0.049	15.183	1	0.000	1.211	1.100	1.333
	Mixed tenure	0.104	0.058	3.160	1	0.075	1.109	0.989	1.243
	N/A	0.477	0.321	2.211	1	0.137	1.611	0.859	3.022
Respondent role	Retired/semi-retired			17.288	7	0.016	1		
	Sole/primary farmer	0.059	0.182	0.106	1	0.745	1.061	0.742	1.516
	Farming member of farm household	-0.031	0.183	0.029	1	0.865	0.969	0.677	1.389
	Non-farming member of farm household	-0.192	0.201	0.913	1	0.339	0.825	0.556	1.224
	Farm employee (director/manager)	-0.204	0.199	1.052	1	0.305	0.815	0.552	1.205
	Farm employee (any other worker)	0.057	0.238	0.057	1	0.812	1.058	0.664	1.688
	Farm contractor	-0.038	0.280	0.018	1	0.892	0.963	0.556	1.666
	Other	0.150	0.212	0.500	1	0.479	1.162	0.766	1.762
				χ^2	df	*p*			
Overall model evaluation				309.918	31	<.001			

Table C.6 Sub-group n values for mean EQ-5D index values by age for a) females and b) males

Age group (years)	Females	Males
18-24	180	159
25-34	267	421
35-44	288	733
45-54	575	1581
55-64	997	3229
65-74	638	2935
75+	321	1684
All ages	3266	10742

Table C.7 Sub-group n values for EQ-VAS by age for a) females and b) males

Age group (years)	Females	Males
18-24	181	162
25-34	268	424
35-44	290	740
45-54	582	1597
55-64	1000	3254
65-74	646	2961
75+	327	1718
All ages	3294	10856
